# 

**Published:** 1912-02

**Authors:** 


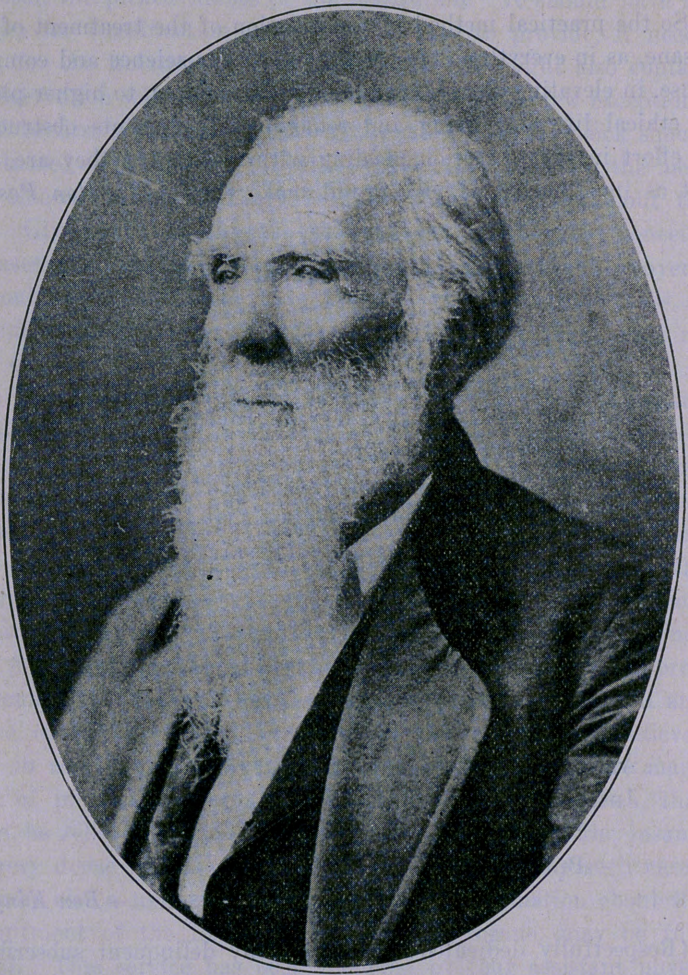

DR. GIDEON LINCECUM.

The most noted pioneer Texas physician and naturalist, author of a famous book on Texas ants, and a work on the indigenous medicinal plants of Texas. His autobiography is preserved in the archives of the Mississippi State Historical Society, and published in Volume VIII of its Transactions. Dr. Lincecum was born in Georgia, April 22, 1793. Died at his home in Washington county, Texas, November 28, 1873, aged 80 years.

